# Editorial: Upper and lower limbs trauma in childhood

**DOI:** 10.3389/fped.2022.1127990

**Published:** 2023-01-10

**Authors:** Vito Pavone, Antonio Andreacchio, Federico Canavese

**Affiliations:** ^1^ Department of General Surgery and Medical Surgical Specialties, University Hospital Policlinico “Rodolico-San Marco”, University of Catania, Catania, Italy; ^2^ Pediatric Orthopedic Surgery Department, “Vittore Buzzi” Children’s Hospital, Milano, Italy; ^3^ Lille University Centre, Jeanne de Flandre Hospital, Department of Pediatric Orthopedic Surgery, Lille, France

**Keywords:** children, fracture, pediatric orthopedic, traumatology, upper extremity, lower extremity

**Editorial on the Research Topic** Upper and lower limbs trauma in childhood

Pediatric Orthopedic Traumatology, as a subspecialty of Orthopedics and Traumatology, has evolved significantly over the past two or three decades and continues to make progress.

Pediatric injuries, due to the rapidly growing skeleton of the patient, have the risk to severely impact lifestyle and mobility despite an extremely favorable healing potential. Therefore, the anatomical features of the child's bone are responsible for the peculiarities of pediatric traumatic injuries and require specific diagnostic methods and treatment options.

The incidence of fractures in pediatric population is about 20 on 1,000 per year, increasing with age; 61% of children's fractures occurs in males (1).

The epidemiology and distribution of pediatric fractures change over time and are influenced by a multitude of factors including geography, climate, and population characteristics (2).

In the pediatric age, fractures occur more frequently than in adults. One reason could be related to children and adolescents that are less skilled at-risk assessment. Furthermore, the skeleton is a dynamically growing system, especially in children; bone is less stable, although more elastic, than in adulthood (3). These properties explain both the higher incidence and, at the same time, the faster healing of fractures in children and adolescents. Treatment of fractures in children requires precise knowledge of the anatomy and growth characteristics of healthy and damaged bones, as well as the specific fracture dynamics in this age group (4). Nevertheless, children are often treated as though they were simply small adults. This leads to imprecise clinical assessment, misinterpretation of the radiographic findings, inappropriate choice of treatment and inadequate follow-up. In the elbow fractures the highest error rate (77%) was reported (5).

The special issue **Upper and Lower limbs Trauma in Childhood** aimed to focus on the diagnostic and therapeutic aspects of some pediatric injuries including subtrochanteric femoral fractures, unstable femoral fractures, avulsion fractures of the pelvis in adolescent athletes and torus fractures.

With regard to diagnostics, the use of computer science is playing an increasingly prominent role. In particular, the dogma that antero-posterior and lateral radiographic projections are necessary to rule out a fracture has been challenged by the advent of new technologies. The use of convolutional neural networks (Janisch et al.) has been shown to rule out the presence of a distal radius torsion fracture. This algorithm could help decrease radiation exposure and patient comfort in the near future without compromising diagnostic quality.

Two other articles review the surgical treatment of pediatric femur fractures. One (Lu et al.) analyzes the role of adjuvant temporary external fixation in diaphyseal fractures of the femur treated with elastic stable intramedullary nailing. The other (Hong et al.) compares the use of triple elastic stable intramedullary nail vs. locking plate in the treatment of subtrochanteric femoral fractures in school-aged children. These two papers highlight how innovative techniques improve the treatment of complex femoral fractures in children. In the first, it is emphasized how the combined use of elastic stable intramedullary nailing and temporary external fixation provides good clinical and radiological outcomes in children with unstable diaphyseal femur fractures aged between 5 and 11 years, with a reduced complication rate (Lu et al.). In the second, it is noted that both triple elastic stable intramedullary nail and locking plate allow satisfactory outcomes in school-aged children with subtrochanteric fractures. However, compared with locking plate, triple elastic stable intramedullary nailing showed significantly less operative time, bleeding and hospital stay (Hong et al.).

Finally, the systematic review on treatment of avulsion fractures of the pelvis in adolescent athletes (Di Maria et al.) provides an overview of all injuries that can occur in young athletes and allows for a review of the diagnosis and treatment of these injuries.

Regarding treatment, interestingly, the authors emphasize the fact that surgery is preferred for major dislocation and fragment size, providing a better return-to-sport rate and decreasing the risk of complications. However, both surgical and conservative treatment provided excellent outcome in most cases.

The Editors of the research topic Upper and Lower Extremity Trauma in Childhood believe that the contents bring new information to a rapidly evolving field of orthopedic trauma.

We hope you enjoy these articles and that they will stimulate further discussion and understanding of pediatric orthopedic traumatology.
